# Success of electrocardioversion on the elderly

**DOI:** 10.1007/s12471-014-0519-y

**Published:** 2014-01-23

**Authors:** J. J. Stroink, N. van Boven, J. H. Ruiter, V. A. W. M. Umans

**Affiliations:** Hart-long Centrum, Medisch Centrum Alkmaar, Postbus 501, 1800 AM Alkmaar, the Netherlands

**Keywords:** Atrial fibrillation, Elderly, ECV, Electrocardioversion, Rate-control, Rhythm control

## Abstract

**Background:**

There are two treatment strategies for medication-resistant atrial fibrillation (AF): rhythm control or rate control. It has been suggested that rate control is a valid strategy in well-tolerated AF because it does not result in increased mortality. We aimed to investigate the 1-year outcome of rhythm control in an elderly population of AF patients.

**Method:**

The study was retrospective, using the data collected from electrocardioversions (ECV) of elderly patients and the data of their follow-up visits to the outpatient clinic. We looked for recurrence of AF within the first year after ECV. Furthermore, we investigated possible predictors of recurrence.

**Results:**

From February 2008 till November 2011, 436 consecutive elderly patients admitted for ECV were included. The 1-year recurrence rate of AF was 51.3 %. We found that being female and a large left atrial diameter were independent predictors of recurrence.

**Conclusion:**

The AF recurrence rate in our elderly population is comparable with reported AF recurrence rates in a younger population; we conclude that rhythm control can be regarded as the viable strategy in persistent AF in elderly patients.

## Background

Atrial fibrillation (AF) is the most common persistent arrhythmia seen by physicians. The incidence of AF increases with age. Population studies have shown a prevalence of AF of 8–10.6 % in populations older than 75 years [1–3]. These numbers are increasing, because of prolonged life expectancy and improved awareness of the condition of today’s mentally and physically healthy elderly population. Two different treatment options are used for persistent AF: rhythm or rate control. In rhythm control, sinus rhythm is maintained or restored with medication and serial electrocardioversion (ECV) or chemical cardioversion. In rate control, particularly used in patients with minor symptoms, AF is accepted and the heart rate, by means of ventricular response, is controlled by medication[4]. However, the recurrence rate of AF after ECV in the elderly population seems disappointingly high, [5,6] as compared with the reported recurrence rate of 40–50 %[7,8] in a younger population. Patients in the rhythm-control treatment group have a smaller chance of progressing to chronic AF[9,10].

Multicentre randomised clinical trials show no difference in morbidity or mortality between asymptomatic patients treated with either rhythm control or rate control[11,12]. The fact that elderly patients have a higher chance of recurrence without a difference in morbidity or mortality between the two strategies suggests that the preferred treatment for the elderly is rate control. However, elderly patients with minor symptoms due to AF have indeed an increased risk of developing heart failure and related admissions[13], which suggests that rhythm control would be the treatment of first choice in these patients. However, current knowledge of predictors for successful rhythm control is limited. Neither echocardiographic nor clinical parameters are powerful predictors[14,15].

Whether rhythm control is a valid treatment option for daily clinical practice among elderly AF patients is not yet determined. We therefore explored the 1-year outcome of all consecutive elderly AF patients who underwent a successful ECV in a large teaching hospital. Specifically, we explored the determinants of AF recurrence, 1-year mortality, number of strokes and major bleeding events.

## Methods

This retrospective cohort study consisted of all consecutive AF patients who were over 75 years of age and had a successful ECV at Medical Centre Alkmaar between February 2008 and November 2011. All patients were treated according to our ECV protocol, which included follow-up visits after 1 and 12 months. This allowed us to perform a 1-year follow-up of all successful ECVs of patients older than 75 years. If patients had another ECV after a recurrence, they were included as a second ECV case. This happened in a few cases. Besides regular visits, we used data from intermediate visits to the emergency room or to other specialities. All data, including patients demographic, clinical, and outcome characteristics, were extracted from the clinical records.

The primary outcomes consisted of: AF recurrence within a month, AF recurrence within 12 months. A recurrence of persistent AF is defined as AF requiring intervention.

The secondary outcomes were: ischaemic events, major bleeding events, all-cause mortality, adequacy of international normalised ratio (INR) during ECV, type of rhythm medication for rate control and whether there was a switch to rate control after recurrence. An ischaemic event was defined as any stroke, transient ischaemic attack or pulmonary embolism. A bleeding event was determined by fatal bleeding or symptomatic bleeding in a critical area or organ, such as intracranial, intraspinal, intraocular, retroperitoneal, intra-articular or pericardial, or intramuscular with compartment syndrome or needing transfusion. INR was considered inadequate if there was more than one measurement below 2 or above 5 within 4 weeks prior to or 4 weeks after ECV.

For the first analysis two groups were used: patients aged 75–79 years and patients aged >80 years. For the second analysis two groups were made: a positive outcome group (no recurrences within a year, no bleeding events, no ischaemic events and no death) and a negative outcome group, i.e. chronic AF recurrence within a year, bleeding events, ischaemic events or death. For the third analysis two groups were used: patients who maintained rhythm control and patients who, after recurrence, were switched to a rate-control strategy.

### Statistics

To compare the characteristics of the different age groups, *P*-values were calculated for all variables using the chi-square test for categorical variables or independent samples *t*-test for continuous variables. Overall statistical significance was set at a two-tailed *P* value <0.05. Cases were labelled as ECV not as patients.

To compare age-related recurrence, log-rank test of the 75–79 years and >80 years group were used to determine the occurrence of the primary endpoint over time.

To determine predictors of a negative outcome, the groups were compared for significant differences using univariate analysis. The test variables were: gender, age, renal function, left atrial diameter and history of heart failure, coronary disease, hypertension, diabetes and rhythm medication used at the time of ECV.

We compared a rhythm-control group with a rate-control group for significance using the appropriate tests already mentioned above in comparison of age groups.

## Results

A total of 477 consecutive successful ECVs were performed in elderly patients from which 41 cases were excluded because of incomplete follow-up. The average age of the remaining 436 patients was 79.6 years and 46.6 % of them were female. Slightly more than 70 % had hypertension and 65 % had a normal left ventricular systolic function (Table 1). Their mean CHA2DS2-VASc and HAS-BLED scores were 4.1 and 3.1 respectively. In the whole study there were 110 (25.3 %) recurrences within a month and 224 (51.3 %) within a year. Seven ischaemic events (1.7 %), 16 bleeding events (3.8 %) and 22 deaths (5.4 %) occurred (Table 1).

**Table 1:** Baseline characteristics and outcome of total population

Total population	*n* = 436
Baseline characteristics	
Mean age	79.63
Female (%)	203 (46.6)
Mean CHA2DS2-VASc	4.13
Mean HAS-BLED	3.1
Coronary disease (%)	134 (30.7)
Diabetic (%)	60 (13.8)
Hypertension (%)	320 (73.4)
History of heart failure (%)	94 (21.6)
Mean potassium	4.41
Mean creatinine	107.68
Mean GFR	52.95
Mean haemoglobin	8.6
Mean LA diameter	45.2
Normal EF (%)	278 (65.3)
Reduced EF (%)	105 (24.6)
Poor EF (%)	43 (9.9)
Outcome	
Recurrences 1 month (%)	110 (25.3)
Recurrences 1 year (%)	224 (51.4)
Stroke 1 year (%)	7 (1.7)
Bleed 1 year (%)	16 (3.8)
Death 1 year (%)	22 (5.4)
Negative outcome (%)	238 (54.6)
Rate control (%)	161 (41.6)
Inappropriate INR (%)	91 (21.9)

*EF* ejection fraction; *GFR* glomerular filtration rate; *INR* international normalised ratio; *LA* left atrial

### Age-related outcome

**Fig. 1:**
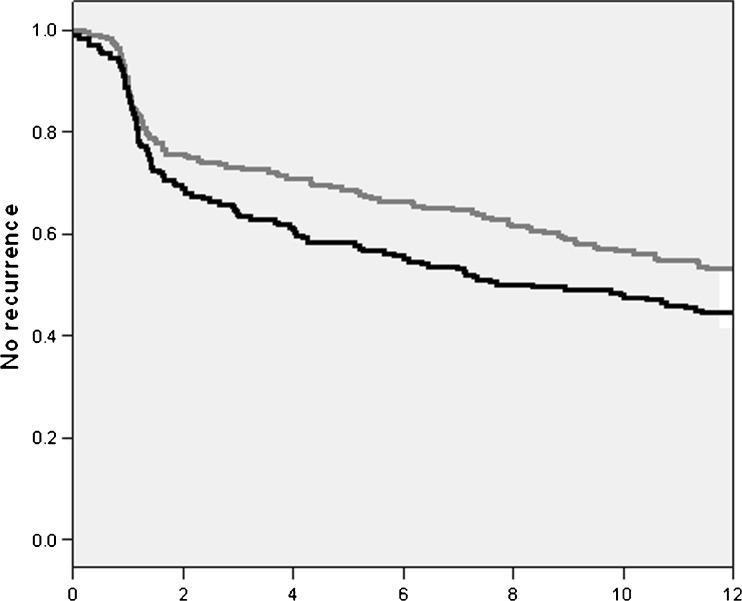
Kaplan-Meier curve, every event is a recurrence. *Grey line*: Group 1: 75–79 years, *black line*: group 2: ≥80 years. *P* = 0.2, timeline is in months

ECV was performed in 229 (52.5 %) patients aged 75–79 and in 207 (47.5 %) patients ≥80 years. Their mean ages were 76.9 and 82.7 years respectively with no patients >91 years, and 55.9 % and 50.7 % respectively were male. Univariate analysis showed no differences between the two groups with respect to gender, cardiovascular risk factors, history of heart failure, left ventricular function and renal function. Mortality and recurrence rates of AF at 1 year were comparable between the two groups (Fig. 1).

### Predictors of ECV success in the elderly

There were 238 patients in the negative outcome group and 198 patients in the positive outcome group. The groups show no significant differences in age, hypertension, mean glomerular filtration rate (GFR) or percentage of patients with a normal left ventricular ejection fraction. But there were more females in the negative group (50.8 % vs. 41.4 %; *p* = 0.05) and more patients with a larger left atrial diameter in the negative group (45.77 vs. 43.99; *p* = 0.03). There were no significant differences in use of antiarrhythmic medication.

### Rhythm vs rate control

For this final analysis 36 more cases were excluded because follow-up treatment after recurrence could not be determined. The first group, the rhythm-control group, contained 236 cases and in the second group, the rate-control group, there were 164 cases. The mean age of the patients in the first group was 79.8 and in the second group 79.6. In the first group 44.5 % were female and in the second group 50.6 %. And 61 % of the patients in the first group had a normal left ventricular function compared with 67.7 % of patients in the second group. None of the characteristics mentioned differed significantly between the two groups. The only baseline difference between the rhythm and rate-control group was mean GFR (54.3 vs. 51.02; *p* = 0.02). Baseline mean creatinine was not different between the two groups (105.5 vs. 110.9; *p* = 0.23) (Table 2).

**Table 2:** Comparing baseline characteristics and outcome between patients with a rhythm-control strategy and patients on a rate-control strategy

	Rhythm control	Rate control	*p*
	*n* = 236	*n* = 164	
Baseline characteristics			
Mean age	79.8	79.6	0.54
Female (%)	105 (44.5)	83 (50.6)	0.23
Coronary disease (%)	79 (33.5)	48 (29.3)	0.07
Diabetic (%)	34 (14.4)	19 (11.6)	0.1
Hypertension (%)	173 (73.3)	122 (74.4)	0.63
History of heart failure (%)	48 (20.3)	36 (22)	0.44
Mean potassium	4.5	4.4	0.62
Mean creatinine	105.5	110.9	0.23
Mean GFR	54.3	51.02	0.02
Mean haemoglobin	8.8	8.5	0.58
Mean LA diameter	44.02	46.47	0.001
Normal EF (%)	144 (61)	111 (67.7)	0.22
Reduced EF (%)	61 (25.8)	35 (21.3)	0.27
Poor EF (%)	24 (10.2)	15 (9.1)	0.71
Outcome			
Recurrences 1 month (%)	29 (12.3)	60(36.6)	0.001
Recurrences 1 year (%)	65 (27.5)	119 (72.6)	0.001
Stroke 1 year (%)	3 (1.3)	3 (1.8)	0.66
Bleed 1 year (%)	3 (1.3)	9 (5.5)	0.02
Death 1 year (%)	9 (3.8)	7 (4.3)	0.87
Inappropriate INR (%)	49 (20.8)	31 (18.9)	0.67

*EF* ejection fraction; *GFR* glomerular filtration rate; *INR* international normalised ratio; *LA* left atrial

More bleeding events were seen in the rate-control group (5.5 %) compared with the rhythm-control group (2.1 % *p* = 0.02). There was no significant difference in the number of cases with an inappropriate INR (*p* = 0.92) (Table 2).

## Discussion

In this retrospective study data collected from ECVs of patients older than 75 years were used to assess the AF recurrence rate in the elderly patient. We tried to find predictors for recurrence, events or death. And a comparison was made of baseline characteristics between patients maintaining sinus rhythm and patients who, after recurrence, were assigned to rate control.

A 1-year recurrence rate of AF of circa 50 % was found. This was not different for patients between 75 and 80 years of age and patients older than 80 years. Nearly half of the recurrences occurred within the first month. This result is not different from the results of studies of the general population[7,8]. This could mean that either the recurrence in an elderly population is comparable with a younger population or that this outcome could has been affected by selection bias. There is a higher chance that the cardiologist chooses a rhythm-control strategy for a healthier patient and vice-versa. This is also evident in the Kaplan-Meier curve in which there is no significant difference but a trend for older patients to have a higher recurrence. This is also reported in the literature [5,6].

Female gender and left atrial diameter were independent predictors of negative outcome. These are known predictors for negative outcome. Other predictors were not found; however, our study was relatively small. Future studies may give a more decisive conclusion on predictors of AF recurrence after ECV in the elderly.

Our 1-year analysis showed no significant differences in mortality and ischaemic events in patients in rate control or in rhythm control. This means that the conclusions of the RACE trial[7], in which no difference in mortality was found in a general population between a rate-control strategy and a rhythm-control strategy, is also true for an elderly population. Even though there was no difference in mortality, there were significantly more bleeding events in the rate-control group. There was no difference in inappropriate INR. This could mean that elderly patients in rate control are either more prone to bleeding events or that the significantly lower GFR was at fault here.

Even though there was no significant difference in mortality between patients in rhythm control and patients in rate control, there could be a higher comorbidity burden in the elderly. Left ventricular hypertrophy will be more common and elderly patients will have worse renal function and more frailty. The maintenance of atrioventricular synchrony is more important in the elderly patient with less cardiac reserve and more pronounced diastolic dysfunction. If they lose their atrial kick, the diastolic filling is even less efficient resulting in heart failure [16–18]. The difference in severity of perceived heart failure symptoms and quality of life is difficult to determine in a retrospective study. It would be interesting for future prospective studies to compare quality of life for elderly patients in either rate control or rhythm control.

## Conclusion

From our study of ECVs in patients older than 75 years with medication-resistant AF three conclusions can be drawn. First of all, the recurrence rate of AF in elderly patients with medication-resistant AF is comparable with younger patients. Secondly, female gender and left atrial diameter were independent predictors of negative outcome. These predictors were also predictors of negative outcome in a population of younger AF patients. Finally, there was no significant difference in mortality in elderly patients in either rhythm control or in rate control. This would suggest that in the elderly a choice between the two strategies should be made purely for reduction of symptoms and not for mortality reduction.
